# Sentinel Lymph Node Mapping in Lung Cancer: A Pilot Study for the Detection of Micrometastases in Stage I Non-Small Cell Lung Cancer

**DOI:** 10.3390/tomography10050058

**Published:** 2024-05-15

**Authors:** Gaetano Romano, Carmelina Cristina Zirafa, Fabrizia Calabrò, Greta Alì, Gianpiero Manca, Annalisa De Liperi, Agnese Proietti, Beatrice Manfredini, Iosè Di Stefano, Andrea Marciano, Federico Davini, Duccio Volterrani, Franca Melfi

**Affiliations:** 1 Minimally Invasive and Robotic Thoracic Surgery, Department of Surgical, Medical, Molecular Pathology and Critical Area, University Hospital of Pisa, 56124 Pisa, Italy; gaetano.romano@ao-pisa.toscana.it (G.R.); f.calabro5@studenti.unipi.it (F.C.); beatrice.manfredini91@gmail.com (B.M.); f.davini@ao-pisa.toscana.it (F.D.); franca.melfi@unipi.it (F.M.); 2 Pathological Anatomy, Surgical, Medical, Molecular, and Critical Care Pathology Department, University Hospital of Pisa, 56124 Pisa, Italy; greta.ali@gmail.com (G.A.); agneseproietti@gmail.com (A.P.); iose.distefano@phd.unipi.it (I.D.S.); 3 Nuclear Medicine, Department of Translational Research and New Technology in Medicine, University of Pisa, 56124 Pisa, Italy; giamanca@gmail.com (G.M.); andrea.marciano2305@gmail.com (A.M.); duccio.volterrani@med.unipi.it (D.V.); 4 2nd Radiology Unit, Department of Diagnostic Imaging, University Hospital of Pisa, 56124 Pisa, Italy; a.deliperi@ao-pisa.toscana.it

**Keywords:** OSNA, sentinel lymph node, NSCLC, robotic surgery

## Abstract

Lymphadenectomy represents a fundamental step in the staging and treatment of non-small cell lung cancer (NSCLC). To date, the extension of lymphadenectomy in early-stage NSCLC is a debated topic due to its possible complications. The detection of sentinel lymph nodes (SLNs) is a strategy that can improve the selection of patients in which a more extended lymphadenectomy is necessary. This pilot study aimed to refine lymph nodal staging in early-stage NSCLC patients who underwent robotic lung resection through the application of innovative intraoperative sentinel lymph node (SLN) identification and the pathological evaluation using one-step nucleic acid amplification (OSNA). Clinical N0 NSCLC patients planning to undergo robotic lung resection were selected. The day before surgery, all patients underwent radionuclide computed tomography (CT)-guided marking of the primary lung lesion and subsequently Single Photon Emission Computed Tomography (SPECT) to identify tracer migration and, consequently, the area with higher radioactivity. On the day of surgery, the lymph nodal radioactivity was detected intraoperatively using a gamma camera. SLN was defined as the lymph node with the highest numerical value of radioactivity. The OSNA amplification, detecting the mRNA of CK19, was used for the detection of nodal metastases in the lymph nodes, including SLN. From March to July 2021, a total of 8 patients (3 female; 5 male), with a mean age of 66 years (range 48–77), were enrolled in the study. No complications relating to the CT-guided marking or preoperative SPECT were found. An average of 5.3 lymph nodal stations were examined (range 2–8). N2 positivity was found in 3 out of 8 patients (37.5%). Consequently, pathological examination of lymph nodes with OSNA resulted in three upstages from the clinical IB stage to pathological IIIA stage. Moreover, in 1 patient (18%) with nodal upstaging, a positive node was intraoperatively identified as SLN. Comparing this protocol to the usual practice, no difference was found in terms of the operating time, conversion rate, and complication rate. Our preliminary experience suggests that sentinel lymph node detection, in association with the accurate pathological staging of cN0 patients achieved using OSNA, is safe and effective in the identification of metastasis, which is usually undetected by standard diagnostic methods.

## 1. Introduction

Lung cancer is the leading cause of cancer-related death worldwide. Low-dose CT (LDCT) screening programs can detect early-stage lung cancer and reduce mortality [1,2,3,4].

Surgery is considered the be the gold standard treatment for patients with early-stage non-small cell lung cancer (NSCLC) and is considered curative. The average 5-year survival rate for patients with completely resected stage I NSCLC is about 70%, with recurrences in 30% of patients [5,6]. The relapse at distant sites is supposed to originate from micrometastases, which are undetected by standard diagnostic methods [7,8]. Indeed, tumor cells in the regional lymph nodes have been detected at an incidence between 20 and 30% and seem to predict shorter disease-free periods and overall survival [9,10,11,12,13].

The reasons for non-detection can be identified as follows:

1. Inadequate lymph node dissection or incomplete removal of the lymph nodes draining the primary tumor [14].
2. Inadequacy of definitive staging due to the sensitivity limits of the methods used to detect the presence of neoplastic cells in the lymph node tissue [14],
3. Presence of “skip-metastases”: the lymphatic drainage of the primary tumor does not always follow the expected pattern, given the possibility that tumor cells exceed the regional lymph nodes and metastasize in distant stations.

Accordingly, it is mandatory to identify patients with nodal metastases who may benefit from adjuvant therapies, as demonstrated by several randomized clinical trials [15,16].

The extension of lymphadenectomy in clinical stage I NSCLC has been controversial due to the lower rate of mediastinal node involvement detected by pathological analysis and the risk of postoperative complications associated with the surgical procedure, including arrhythmia, neurological injury, prolonged air leak, pneumonia, atelectasis, chylothorax and bronchopleural fistula [17,18]. The detection of sentinel lymph nodes (SLNs) during the surgical operations of lung cancer can represent a strategy to minimize the impact of the technical procedure, ensuring adequate staging and treatment.

The sentinel lymph node is defined as the first lymph node (or group of lymph nodes) that receives afferent lymphatic drainage from a primary tumor. Several studies concerning the usefulness of SLN in NSCLC have been reported, showing that systematic SLN mapping could allow surgeons to target the first tumor-draining lymph node for sampling and in-depth pathologic analysis. To date, the debate on its routine use in patients who undergo anatomical resection for lung cancer is still open [19,20,21,22].

One-step nucleic acid amplification (OSNA), a rapid intraoperative molecular detection technique, has recently been confirmed as effective in the intraoperative diagnosis of lymph node metastases in non-small cell lung cancer. Although there are studies on the performance of sentinel node detection using OSNA in breast cancer, no previous studies have evaluated the combination of these techniques in lung cancer.

Our pilot study aims to evaluate the intraoperative identification of sentinel lymph nodes in early-stage NSCLC, using a novel molecular agent for lymphatic mapping ([99mTc]-tilmanocept) [23], with the assessment of lymph node metastases and N2 “skip metastases” by OSNA (one-step nucleic acid amplification) analysis [24] to optimize intraoperative lymph node staging.

## 2. Materials and Methods

Consecutive patients undergoing pulmonary resection with lymphadenectomy for NSCLC were enrolled from March to July 2021. All patients underwent intraoperative sentinel lymph node identification and subsequent analysis of the removed lymph nodes by OSNA. All surgical procedures were conducted with a four-arm robotic technique using the da Vinci Xi surgical system (Intuitive SRL, Sunnyvale, CA, USA).

Concerning pathological staging, the 8th Edition of the TNM of the International Association for the Study of Lung Cancer (IASLC) was used; histological diagnoses and pathological features were obtained by two pathologists (G.A. and A.P.) according to the WHO 2021 histological and immunohistochemical criteria (WHO Classification of Tumours, Thoracic Tumours, 5th Edition, Volume 5. International Agency for Research on Cancer, 2021).

Each patient signed an informed consent form before surgery to be included in the study.

Routine preoperative staging was performed, including clinical examination, blood analysis, a standard chest X-ray, head/thoracic/abdomen computed tomography (CT), positron emission tomography/computed tomography (PET/CT) with 18fluorine-fluorodeoxyglucose (18F-FDG), respiratory function tests (spirometry and diffusing capacity of the lung for carbon monoxide), cardiological and anesthetic evaluation.

### 2.1. Eligibility

The inclusion criteria for enrollment in the study were as follows: diagnosis of adenocarcinoma or squamous cell carcinoma of the lung; clinical stage IA-IB; no evidence of lymphadenopathy on preoperative staging investigations (cN0); adequate bone marrow function; adequate respiratory function defined by an FEV1 greater than 50% of the predicted value; informed consent signature; and age over 18 years.

### 2.2. Technique

#### 2.2.1. TIME 0: Lesion marking and SPECT Analysis

The day before surgery, patients underwent CT-guided marking of the primary lung lesion followed by single-photon emission computed tomography (SPECT). CT-guided lesion marking was performed for all patients in the supine position with a 22G fine needle. During the procedure, a specific radiopharmaceutical for lymphoscintigraphy, consisting of [99mTc]-tilmanocept (Lymphoseek^®^), was injected into the tumor; each administration was made with a double injection, starting in the central part and then moving to the peripheral part of the lesion. Tilmanocept (Lymphoseek^®^) is a synthetic nanomolecule with a size of approximately 7 nm and a molecular weight of 19 kDa. It comprises multiple units of mannose and diethylene triamine penta-acetic acid (the labeling site for [99mTc]), attached to a 10 kDa dextran backbone [25]. This design is aimed at targeting mannose-binding receptors (CD 206) on the surface of lymphatic resident reticuloendothelial cells.

All patients received 18 Mbq of 99mTc-tilmanocept at a total volume of 10 mL.

**Figure 1:**
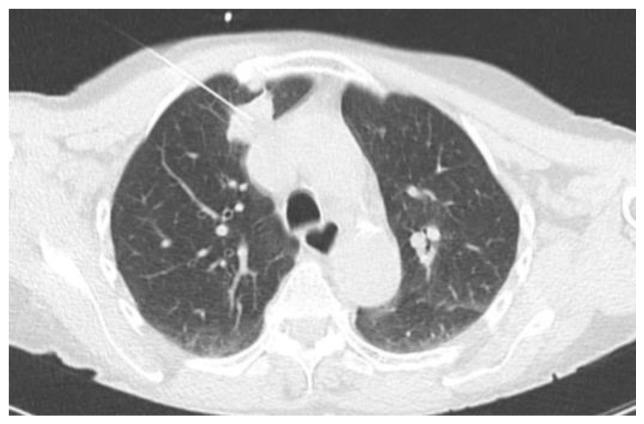
CT-guided injection of the [99mTc]-tilmanocept.

**Figure 2:**
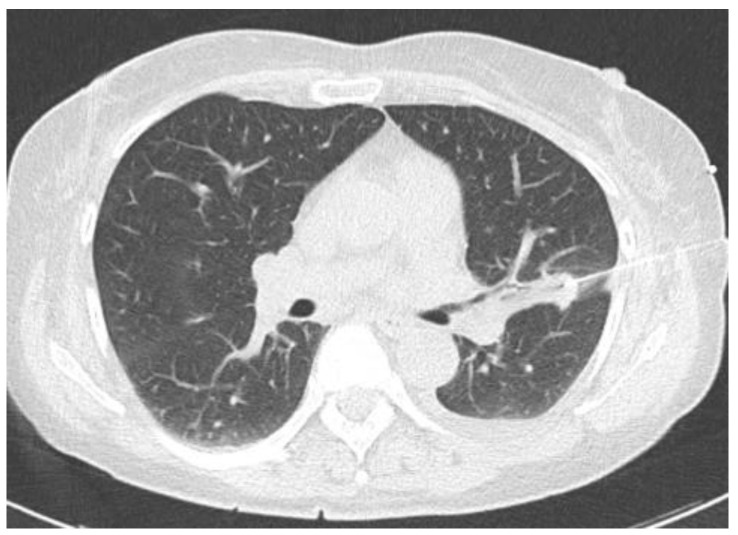
CT-guided injection of the [99mTc]-tilmanocept.

Two examples of CT-guided radiopharmaceutical injection are shown in Figure 1 and Figure 2.

At the end of the procedure, all patients underwent follow-up tomographic scans to check the radiopharmaceutical distribution and detect any possible signs of post-procedural complications.

**Figure 3:**
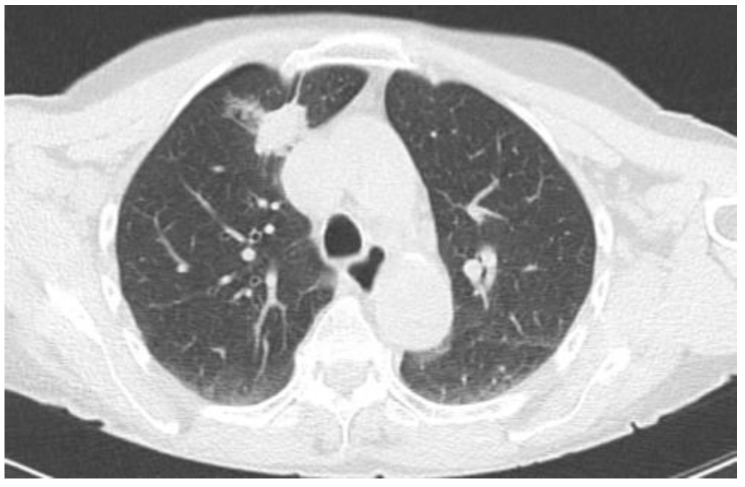
CT follow up after radiopharmaceutical injection.

**Figure 4:**
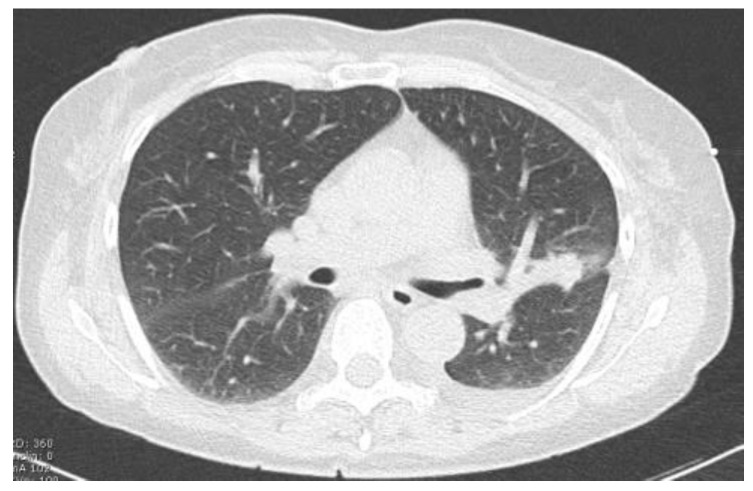
CT follow up after radiopharmaceutical injection.

Two examples of control CT scans are shown in Figure 3 and Figure 4 (the images refer to the patients shown in Figure 1 and Figure 2).

**Figure 5:**
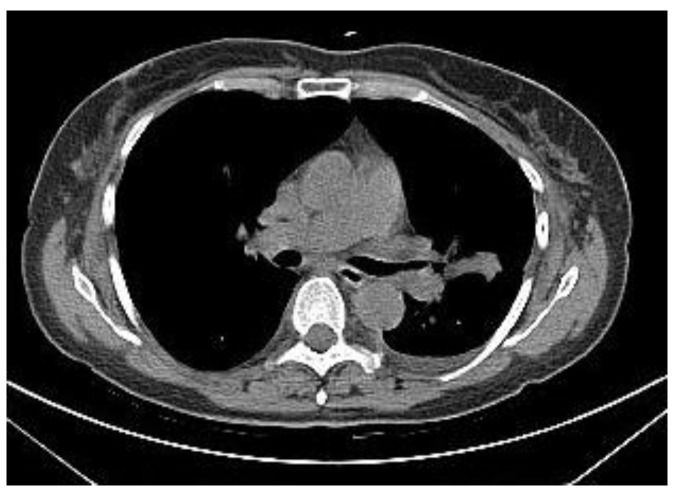
CT scan identification of a hilar lymph node as a sentinel lymph node.

**Figure 6:**
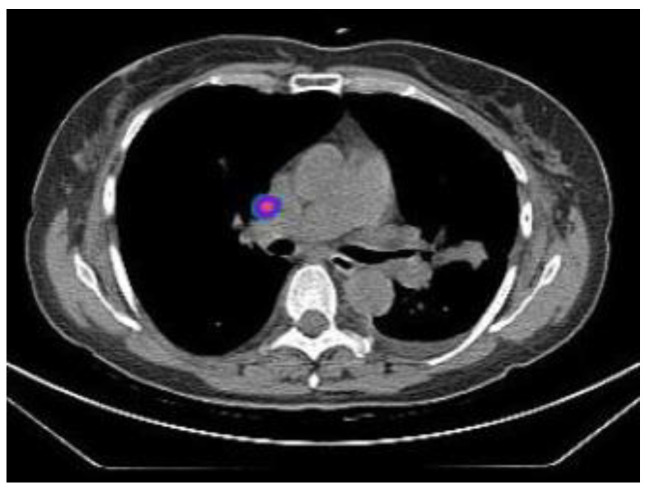
SPECT scan identification of a hilar lymph node as a sentinel lymph node.

Following this, all patients underwent SPECT/CT (single-photon emission computed tomography with computed tomography) for precise anatomical localization of the sentinel nodes (Figure 5 and Figure 6). Images were acquired at 3.56 ± 0.85 (2.27–4–63) h after injection using a dual/head SPECT/CT gamma camera (Discovery 670, General Electric Healthcare, Waukesha, WI, USA). The following SPECT parameters were used: a low-energy high-resolution collimator, 128 × 128 matrix, zoom factor 1.0, with 120 projections over 360°, 180° per detector, and a step of 3°, 40 s/projection. CT (140 Kv, 40 mAs, 3.75 mm thickness) was used for attenuation correction and anatomical localization. SPECT, CT, and fused images were displayed in axial, coronal, and sagittal planes, and tridimensional maximum intensity projection (MIP).

#### 2.2.2. TIME 1 Surgery

On the day of surgery, each patient underwent lung resection and LN removal with robotic techniques using the da Vinci Xi system (Intuitive Surgical, Sunnyvale, CA, USA).

The intraoperative detection of the activity levels of the radiotracer was executed using a manual probe for the detection of gamma rays (Scinti Probe^®^ MR100, Pol.Hi.Tech., L’Aquila, Italy).

The uptake level was initially detected at the injection site and lymph node station 10 (hilum).

Afterward, mediastinal dissection was performed for the in vivo detection of the radioactivity signal at this level. The radioactivity detected for each LN level was transcribed to identify the LN level with the greatest numerical value. Complete lymphadenectomy was performed in all cases after the detection of radioactivity.

#### 2.2.3. TIME 2: Ex vivo Detection and OSNA Analysis

Subsequently, each lymph node was removed, and its radioactivity was detected ex vivo.

The sentinel lymph node was defined as the individual lymph node for which the probe recorded the highest numerical value of radioactivity or as the group of lymph nodes that showed an increase of >10% in maximum activity after the elimination of the background signal, which was set to a numerical value from 0 to 10.

After the lymph node dissection was completed, a next evaluation with a Scinti Probe of the mediastinal field was performed to detect residual activity. Following this, pulmonary resection was performed.

Within 15 min of sampling, each lymph node was placed in a sterile screw-capped bottle, labeled with the identification number of the lymph node station sampled, and stored on ice until it was taken to the pathology laboratory, as required, for correct OSNA examination. Each lymph node sample was assessed by a pathologist before the analysis as follows: the weight was measured (optimal range for the method 50–600 g), any fatty tissue was removed, and the lymph node was then stored at −80 °C.

The OSNA machine subsequently processed the whole LN lysate. CK19 mRNA automated amplification with a ready-to-use reagent kit (formulated without the necessity of calculation, dilution, or pipetting) was performed directly from the sample lysate. Based on previous studies in breast, gastric, and colorectal cancer patients, LNs were defined as ‘negative’ or ‘positive’ according to established cut-off values [26,27]. Therefore, negative nodes were classified for CK 19 mRNA ccP/μL as less than 250. Isolated tumor cells in LNs were reported as having a ‘late’ rise, i.e., copy numbers rose to <250 copies/mL. LNs positive for metastases (+) showed mRNA CK19 levels of 250–5000 ccP/μL.

## 3. Results

From March 2021 to July 2021, data concerning eight patients undergoing lung resection with a totally endoscopic robotic technique for stage IA-B NSCLC were collected and analyzed.

Our series included 3 female patients (37.5%) and 5 males (62.5%) with a mean age of 66.4 years (range 48–74). Specifically, 2 (25%) patients underwent wedge resections (1 resection of the left lower lobe and 1 of the right upper lobe), 1 (12.5%) patient underwent left S1 + 2 + 3 segmentectomy and 5 patients underwent lobectomies (62.5%), of which 3 cases were right upper lobectomies, 1 was a right lower lobectomy, and 1 was a left upper lobectomy. The average time of surgery was 235 min (range 135–280). Clinical and postoperative details are reported in Table 1.

Sentinel lymph nodes were identified during the surgical procedure in all cases. Details of the detection of the sentinel lymph node are reported in Table 2.

A total of 43 lymph node stations (with a mean of 5.3) were removed during the eight surgical procedures and analyzed with the OSNA assay. All removed lymph nodes were examined with OSNA to assess any pathological positivity of the lymph nodes other than SLNs and consequently assess the sensitivity of the procedure.

No intra or postoperative complications were observed, and the average length of hospital stay was 3.9 days (range 3–6 days).

Histologic examinations resulted in 7 adenocarcinomas (87.5%) and one squamous cell carcinoma (12.5%). According to the 8th edition of the TNM classification, the pathological stage was IA in 2 (25%) patients, IB in 3 (37.5%), and IIIA in 3 (37.5%) cases.

Out of the total 43 lymph node stations analyzed by OSNA, 9 (20.9%) were positive, and lymph node metastasis occurred in 3 (37.5%) out of 8 patients.

The features of lymph nodal involvement in upstaged patients are illustrated in Table 3.

As a result, the pathological examination of the lymph nodes resulted in nodal upstaging in three patients (37.5%), allowing these patients to be moved from clinical stage IB to pathological stage IIIA. In addition, in 2 out of 3 (67%) patients with nodal upstaging, a positive node was intraoperatively identified as a sentinel lymph node (SLN).

## 4. Discussion

Surgical resection with systematic lymphadenectomy is still considered the gold standard for the treatment of resectable NSCLC. However, the removal of lymph nodes is not routinely performed, as evidenced by a survey conducted by the American College of Surgeons, which found that lymphadenectomy was not carried out in more than 40% of patients who underwent lung resection for lung cancer [28]. This practice could be related to several reasons; however, the detection of sentinel lymph nodes during surgery for lung cancer could overcome the problems that lead to the lack of pathological staging of lymph nodes.

The introduction of SLN mapping techniques into surgical practice is relatively recent, dating back to the early 1990s. It has been used for the intraoperative staging of various solid tumors, initially by vital dyes and later with radiotracers, becoming the standard of care for breast cancer and melanoma.

Alex and Krag were the first authors to describe, in 1993, the use of a radioactive tracer (colloid sulfide labeled with 99mTc) for the detection of lymph node radioactivity using a gamma camera [29].

The first study conducted in NSCLC patients was published in 1999. The authors described an SLN detection rate of 47% in lung cancer patients via the intra-operative injection of Isosulfan blue dye at the tumor sites [19]. In 2000, Liptay reported his experience with SLN detection using an intraoperative tumor injection of filtered 99mtechnetium colloid (the radionuclide method) and subsequent lymph node identification using a gamma-ray detector. In this case, the detection rate of SLNs was 82%, demonstrating the higher accuracy of radiotracer-based techniques compared to vital dye-based techniques [18].

Nomori and colleagues first reported the single-shot preoperative peritumoral injection of the 99mtechnetium “tin” colloid using a CT-guided technique, followed by the intraoperative detection of SLNs with a gamma probe 25. SLN identification rates have reached 80% since this experience and subsequent studies [29,30]

Indeed, studies performed at our institute have shown a similar identification rate [20,21,22,23,31]. In 2003, we published our preliminary experience with SLN detection in 26 consecutive patients with resectable NSCLC [20]. The intraoperative injection of a 99mTc nanocolloid at the periphery of the tumor was performed in the first ten patients, while the following patients were injected under computed tomography guidance. SLNs were identified in 25/26 (96.1%) of patients, of which 22.5% were positive for metastatic involvement by histological examination and IHC.

In a later experience, 22 consecutive NSCLC patients with stage I disease underwent sentinel lymph node mapping [32]. RT-PCR analysis for cytokeratin 7 and 19 (CK7-CK19) was used to identify tumor-derived material in the lymph nodes after their detection by the peri-lesional injection of a 99mTc nanocolloid suspension. Each SLN was cut in half as follows: one half was used for conventional examination (H&E staining/by immunohistochemistry (IHC), and the other half was snap-frozen at −80 °C for the RNA detection of CK7 and CK19. SLNs were detected in 16 out of 19 patients.

Positive findings in breast cancer and melanoma have shown that sentinel lymph node mapping is a sensitive and specific method for the following:

1. The identification of micrometastases (“ultrastaging”);
2. The identification of “skip metastases”;
3. Choosing the extent of surgical resection.

In breast cancer, the use of SLN mapping has completely replaced systematic radical lymphadenectomy procedures, allowing a reduction in operating time and postoperative complications, particularly in early-stage patients. In NSCLC, by contrast, the role of SLN remains controversial due to the great anatomical variability of mediastinal lymphatic drainage and the low specificity of the methods in use for frozen section analysis.

Due to its characteristics, OSNA is effective in the intraoperative diagnosis of lymph node metastases in non-small cell lung cancer. Although there are studies on the performance of sentinel node detection using OSNA in breast cancer, no previous studies have evaluated the combination of these techniques in lung cancer. Our study evaluates sentinel lymph node detection in lung cancer using [99mTc]-tilmanocept in association with OSNA analysis, which can detect lymph node micrometastases, aiming to increase the sensibility of the procedure.

In our experience, characterized by the use of [99mTc]-tilmanocept to detect the sentinel lymph nodes in the proximity of the lung tumor, we were able to identify the sentinel lymph node in 100% of cases without procedural-related complications. [99mTc]-tilmanocept is a receptor-binding molecular imaging agent specifically designed to target mannose-binding receptors on the surface of lymphatic resident reticuloendothelial cells, predominantly macrophages and dendritic cells. This agent has demonstrated excellent performance in staging various solid tumors [23], including melanoma [33,34], breast cancer [33,35], and squamous cell carcinoma of the oral cavity [33,36].

Owing to its structural and molecular properties, [99mTc]-tilmanocept potentially offers several advantages over conventional 99mTc-radiocolloids [31]. These include specific receptor lymph node binding (CD 206), rapid clearance from the injection site, high efficiency in sentinel node extraction, and minimal accumulation in higher echelon (station) nodes, resulting in the more accurate detection of sentinel nodes close to the primary tumor [37].

In our series, following the identification of the sentinel lymph node, radical lymphadenectomy was performed in all patients to assess the possible presence of lymph node metastases in stations that are not the sentinel lymph nodes, identifying skip metastases. The pathological analysis revealed metastases in the lymph nodes in three cases, with the sentinel lymph node involved with tumor cells in two patients. In the other patient with metastatic lymph nodes, the positive lymph node station was a skip metastasis in station 9, while the sentinel lymph node was in station 11. The rate of skip metastasis could reach about 30% according to previous studies, and this is in line with our experience [38,39]. In lung cancer, skip metastasis clearly showed how difficult it was to predict the lymphatic drainage of different areas of the lung.

However, the application of new techniques characterized by a high specificity for the intraoperative pathological analysis in lung cancer, such as the OSNA assay, could detect metastatic lymph nodes during sentinel lymph node mapping, thus addressing a more extended lymphadenectomy.

Currently, the standard histopathological examination involves cutting lymph nodes into very thin histological sections, which, after fixation and paraffin embedding, are stained with haematoxylin and eosin. However, this method can fail to detect micrometastatic clusters of cancer cells or isolated cancer cells, such as cells outside the examined section, which can occur in up to 20% of cases [40]. Over the years, several studies have documented up to 20–30% false–negative results using standard histopathological techniques [41,42,43,44,45]. Therefore, failing to detect micrometastases may result in downstaging neoplastic disease. Furthermore, the histology-based technique is time-consuming and can be prone to operator errors.

The most recent approach to detect micrometastases in lymph nodes is a molecular genetic technique known as one-step nucleic acid amplification (OSNA) [44,45], a rapid and semiquantitative assay based on a reverse transcriptase-dependent isothermal amplification reaction (RT-LAMP) for the intraoperative assessment of CK-19 mRNA and its expression in lymph nodes, analyzing the whole lymph node. In lung cancers, such as adenocarcinoma and squamous cell carcinoma, which are characterized by high CK19 expression in approximately 95% of cases, OSNA has been successfully used to diagnose lymph node metastases. This is due to its diagnostic sensitivity, which is based on the maintenance of CK19 expression between the CK19-positive lung site and the corresponding metastatic lymph nodes [46,47,48,49,50,51]. However, OSNA is not indicated for patients diagnosed with carcinoid or pleomorphic tumors, as these tumors express low levels of CK19.

In our series, we observed a high rate of nodal upstaging, which may be related to the ability of OSNA analysis to detect micrometastases, which are not easily diagnosed by standard pathological analysis. The high diagnostic accuracy of OSNA has opened up new diagnostic and staging perspectives in NSCLC patients, such as the use of this assay to provide a rapid assessment of lymph node status during segmentectomy or to identify sentinel lymph nodes [52,53].

## 5. Conclusions

In this pilot experience, the use of [99mTc]-tilmanocept facilitated lymphatic mapping and sentinel node detection with a high success rate. The implementation of sentinel lymph node detection with the OSNA assay may open up new scenarios in the surgical treatment of lung cancer. The OSNA assay has demonstrated significant results in the diagnosis of metastatic lymph nodes in patients with early-stage NSCLC undergoing minimally invasive surgery. Prospective studies with larger numbers of patients and comparative studies are mandatory.

## Figures and Tables

**Table 1:** Details of clinical and postoperative outcomes.

	Pt 1	Pt 2	Pt 3	Pt 4	Pt 5	Pt 6	Pt 7	Pt 8
Sex	F	M	M	F	M	F	M	M
Age	61	66	65	73	70	74	71	48
Smoker/previous smoker	No	Yes	Yes	No	No	Yes	Yes	Yes
Charlson index	5	4	2	4	2	2	5	2
Site of lung cancer	LUL	RLL	RUL	LLL	RUL	RUL	LUL	RUL
Clinical stage	IB	IB	IB	IA	IA	IB	IB	IB
Histotype	ADC	ADC	ADC	ADC	ADC	ADC	SCC	ADC
Postoperative length of stay	4	6	4	3	3	4	4	3
Postoperative complications	No	No	No	No	No	No	No	No
Sentinel lymph node detection	+	+	+	+	+	+	+	+

ADC adenocarcinoma; SCC squamous cell carcinoma; + positive detection.

**Table 2:** Details of sentinel lymph node detection.

	Site of Lung Cancer	Histotype	SPECT-Positive SLN Station	Intraoperative Positive SLN Station
Patient 1	LUL	ADC	10	10
Patient 2	RLL	ADC	*	9
Patient 3	RUL	ADC	11	11
Patient 4	LLL	ADC	#	11
Patient 5	RUL	ADC	#	4
Patient 6	RUL	ADC	10	10
Patient 7	LUL	SCC	*	5
Patient 8	RUL	ADC	10	10

* No lymph node positivity. # SPECT not carried out.

**Table 3:** Characteristics of lymph node involvement in upstaged patients.

	Surgical Procedure	Histotype	Number of Positive LN Stations	Rate of Positive LN Stations	Level of Positive LN Station (s)
Patient 1	LUL	Adenocarcinoma	4	80%	5, 6, 7, 10
Patient 2	RLL	Adenocarcinoma	4	80%	4, 7, 9, 11
Patient 3	RUL	Adenocarcinoma	1	14%	9

## Data Availability

The data underlying this article will be shared by the corresponding author upon reasonable request.
